# A Trajectory Compensation Method Considering the Car-Following Behavior for Data Missing of Millimeter-Wave Radar in Roadside Detection Applications

**DOI:** 10.3390/s23031515

**Published:** 2023-01-30

**Authors:** Rui Zhang, Haiqing Liu, Kunmin Teng

**Affiliations:** 1 College of Transportation, Shandong University of Science and Technology, Qingdao 266590, China; 2 School of Transportation and Logistics Engineering, Shandong Jiaotong University, Jinan 250399, China; 3 College of Electrical Engineering and Automation, Shandong University of Science and Technology, Qingdao 266590, China

**Keywords:** traffic detection, millimeter-wave radar, missing data compensation, car-following model

## Abstract

Concerning roadside traffic detection applications, and to address the millimeter-wave radar’s missing data problem caused by target occlusion or the absence of features in low-speed conditions, this paper proposes a trajectory compensation method regarding car-following behavior. Referring to the installation scheme of the detector, a coordinate transformation method is presented to unify the radar spatial coordinates with the road coordinates. Considering the driver’s car-following behavior, the optimal velocity model (OV), full velocity difference model (FVD), and the full velocity difference and acceleration (FVDA) model are applied for tracking the vehicle’s trajectory related to the movement of the vehicle ahead. Finally, a data compensation scheme is presented. Taking actual trajectory data as samples, the proposed methods are verifiably useful for compensating for missing data and reconstructing target trajectories. Statistical results of different missing data trajectories demonstrate the rationality of the application of car-following models for the missing data compensation, and the FVDA model performs well compared with the OV and FVD models.

## 1. Introduction

Real-time and accurate road traffic parameter collection is the prerequisite for applying better traffic control strategies to improve traffic efficiency and decrease the accident rate [1,2,3]. In recent years, the FMCW millimeter-wave radar has been widely implemented for tracking multi-target trajectories in roadside installation scenarios. This radar can obtain a vehicle’s global moving continuity features compared to embedded detectors (such as loop and geomagnetic detectors) [4,5], which can only detect local cross-section features. It is highly accurate regarding distance and speed, and is minimally affected by environmental fluctuations compared to video devices [6,7]. Given these advantages, the millimeter-wave radar will be one of the most important traffic detectors for traffic flow detection and vehicle behavior recognition in future intelligent transportation systems.

The FMCW millimeter-wave radar generally works at a 30~300 GHz frequency band. The wavelength is from 1~10 mm, and it pays accurate attention to high-speed measurement precision, high multi-objective resolution capacity, imaging quality, wide detection range, and strong anti-interference capability [8,9,10]. However, the Doppler effect decreases [11,12] when the target moves at a certain low-speed, resulting in missing data when the vehicle stops in a waiting line or traffic is congested. Additionally, when the vehicle is closely tailgating a larger target, the trajectory is not continuous due to the loss of signal because of obstruction by the former vehicle. Hence, it makes sense to compensate for the missing data in complex road traffic scenarios.

In the current research, the traditional millimeter-wave radar data compensation methods include historical mean, mode values, and interpolation methods [13]. These methods mine the regulation of the radar data itself and compensate for the missing data concerning the data variation features while neglecting the constraints of the driving behavior between adjacent vehicles. In this paper, a trajectory compensation method concerning car-following behavior is proposed. The optimal velocity model (OV), full velocity difference model (FVD), and full velocity difference and acceleration (FVDA) model are used to describe the driver’s car-following behavior and further track the vehicle’s trajectory for data compensation purposes.

The rest of the paper is organized as follows. Section 2 presents the related literature. In Section 3, the experimental scenario and the missing radar data issue are presented. In Section 4, the trajectory compensation method regarding the car-following behavior is proposed. In Section 5, the data compensation scheme is verified based on the actual road traffic data. Finally, in Section 6, we conclude the paper and discuss future work.

## 2. Related Work

Modern transportation systems rely increasingly on the availability and accuracy of traffic detector data to monitor operational traffic conditions and assess system performance. In practice, the traffic parameters are collected mainly by the magneto-electric induction detecting method [14,15], the floating vehicle method [16,17], the video image detecting method [18], and the radar detecting method. Compared with the other mentioned methods, the radar detection method has the advantages of convenient installation and low interference with traffic flow. The millimeter-wave radar with the 30–300 GHz frequency band and the 1–10 mm wavelength performs well under adverse weather and light conditions. Therefore, it is widely used for automotive vehicles and road traffic management.

Automotive radars are sensors used to provide safety and driving support and are essential to assist the control of autonomous vehicles. Accurate and timely information in the traffic environment is important for stable control. In [19], a vehicle-length estimation method using data from automotive frequency-modulated continuous-wave (FMCW) radar is proposed. Information on the length of vehicles can provide more information for decision-making and be useful for object identification in scenarios such as autonomous driving. If automobiles can identify objects perfectly by using radar systems, it helps prevent accidents. In [20], a human–vehicle classification using a support vector machine (SVM) in a 77-GHz FMCW radar system is proposed. The proposed method shows good classification performance in distinguishing a pedestrian, a vehicle, and a cyclist in practical situations. The radar has been used in urban traffic and expressway management, such as traffic flow volume collection and over-speed detection. In [21], a method of overflow identification and control at intersections is proposed based on millimeter-wave radar data, which is installed at the departure road intersection to detect data. Then, a new intersection phase-timing calculation method is proposed based on the data collected by radar [22].

However, for traffic flow volume collected from complex traffic scenarios, there is inevitably noise or data loss. Datasets, including noise and loss, can lead to suboptimal operations and ineffective decisions. Researchers attempted to evaluate the relationship between original radar data and target, road facilities, geometry, and other traffic environment factors in traffic volume detection [23,24]. These studies have created a solid foundation for better acquiring data and dealing with the problem.

To obtain effective traffic information from the detection environment, many data processing algorithms and filtering are added to the sensor system. Using the Monte Carlo simulation principle constant false alarm rate (CFAR) [25] and the probabilistic data association filter (PDAF) [26], the detection ability of radar for moving targets has improved in the traffic environment. In addition, a two-step abnormal data processing method for millimeter-wave radar in traffic flow detection applications is proposed [27]; suitable thresholds for reducing the number of samples with significant abnormalities in each parameter. In [28], the authors propose a novel approach to improve the detection probability of low-speed small dim targets—converting radar data into two-dimensional images to suppress background noise. The method combines the advantages of millimeter-wave radar and other detectors and also improves the accuracy of the traffic detection system. In [29], millimeter-wave radar and infrared cameras were selected as sensors for vehicle detection. The detection information of millimeter-wave radar can extract the vehicle region of interest (ROI) from infrared images, and infrared images can compensate for the low resolution of the radar. The fusion of radar and image has good complementary advantages for vehicle detection. In [17], an improved incident detection method based on radar–camera fusion is proposed. The detection accuracy is improved by the fusion system. All these methods improve the availability of radar data. However, a multi-sensor information fusion approach can increase the cost of automation.

Although researchers continue their efforts to reduce missing radar data, the issue remains. Therefore, it is important to compensate for lost data collected by sensors. As a simple method for compensation, the value of a lost item can be set as the historical mean, median, and mode value [30,31]. However, simple methods cannot perform well when the amount of missing data is large, especially when they are faced with continuous missing data. The interpolation methods concerning the data collected near the missing data can improve the imputing accuracy and perform better under some practical conditions. In [32], a convex interpolation considering radar accuracy for object vehicle tracking is proposed. The authors analyze radar accuracy by comparing radar and ground truth values in a uniformly divided area. They then use the convex interpolation method based on the radar accuracy analysis to improve radar performance. In [33], authors propose two cokriging methods to impute high-resolution traffic speed data under different data missing pattern scenarios. The radar detector and probe vehicle data are used for the cokriging-based imputation approaches. In [34], a neighboring approximate interpolation method is proposed. Through joint calibration and interpolation, the radar and the camera are angularly aligned in the horizontal direction. The authors achieved the spatial alignment of the heterogeneous data of millimeter-wave radar and camera in the common dimension.

The above methods replace using historical radar data or interpolation to compensate for the missing radar data using relationships between the existing data. However, we argue that previous literature suffers from certain weaknesses. Few studies have investigated compensating for missing radar data using the motion regularity of the detected target itself. Each vehicle driver controls a car under the stimuli from the preceding in the case of traffic operation, and the driver’s car-following behavior can be described as a car-following method. In this paper, a trajectory compensation method that considers the car-following behavior in urban traffic is proposed. This compensation period starts when target data are considered lost, and it ends when the missing data re-emerges in the data set collected by radar. The OV, FVD, and FVDA methods are used to compensate for the missing data during data loss. In our work, the missing data trajectories are completed by the proposed compensation method, and the completed trajectories provide useful support for an intelligent transportation system and its applications.

## 3. Experimental Scenario and Problem Presentation

**Figure 1:**
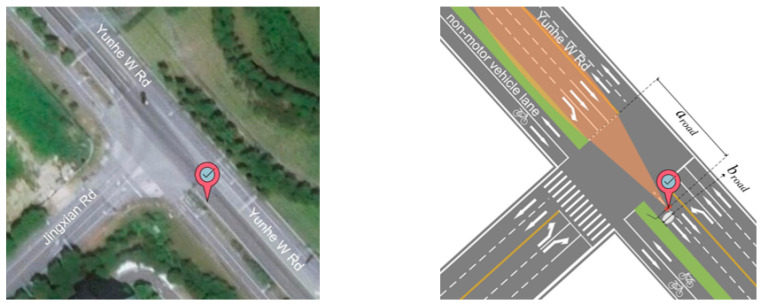
Experimental intersection.

To collect traffic data for further analysis, the experiment is conducted in an actual traffic scenario. The radar device is installed 6–8 m on an upright stanchion alongside the Yunhe Xi road in Wuxi City, China. The location and channelization of the intersection are shown in Figure 1. The detailed parameters of the installation and the road section, together with the technical parameters of the radar device, are introduced in Table 1. In the experiment, the traffic flow of the northwest approach is detected as the data sample, including the vehicle’s radial distance, horizontal distance, radial speed, and horizontal speed per 75 ms outputting the data.

**Figure 2:**
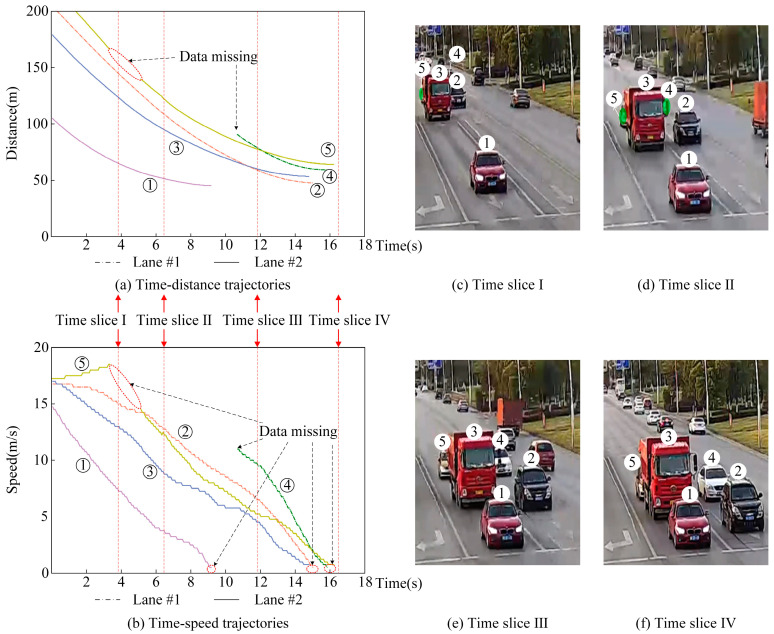
Examples of radar detecting trajectories and data missing problem presentation. (**a**) Time-distance trajectories; (**b**) Time speed trajectories; (**c**) Time slice Ⅰ; (**d**) Time slice Ⅱ; (**e**) Time slice Ⅲ; (**f**) Time slice Ⅳ.

Under the experimental scenario described above, the original data of the road traffic flow are collected. Part of the sampling data is selected and presented in Figure 2.

As shown in Figure 2, vehicle trajectory ④ and ⑤ are interrupted at time slice Ⅰ due to the obstructions from the former vehicle ③. Then, vehicle ④ is detected at time slice Ⅱ, but vehicle ⑤ also blocked until at time slice Ⅲ. Besides, when vehicles stop in a queue at time slice Ⅳ, the data is also missed at a certain low-speed (according to statistics, the speed threshold is about 0.5 m/s in the experiment). Addressing this problem, we propose a data compensation method considering the car-following behavior for obtaining a precise and continuous trajectory in complex road traffic scenarios.

## 4. Data Compensation Method based on Car-Following Behavior

### 4.1. Coordinate Transformation

The actual road environment where the radar is installed is considered to be a three-dimensional space, while the detecting trajectory data is a two-dimensional coordinate system established by the radar antenna horizon plane and the vertical line (i.e., radar coordinate system $O^{radar}$), and the vehicles are driving on the two-dimensional coordinate system established by the lane cross-section and lane line (i.e., road coordinate system, $O^{road}$). Hence, a coordinate transformation model between the radar and road coordinates based on the device installation scheme is needed.

**Figure 3:**
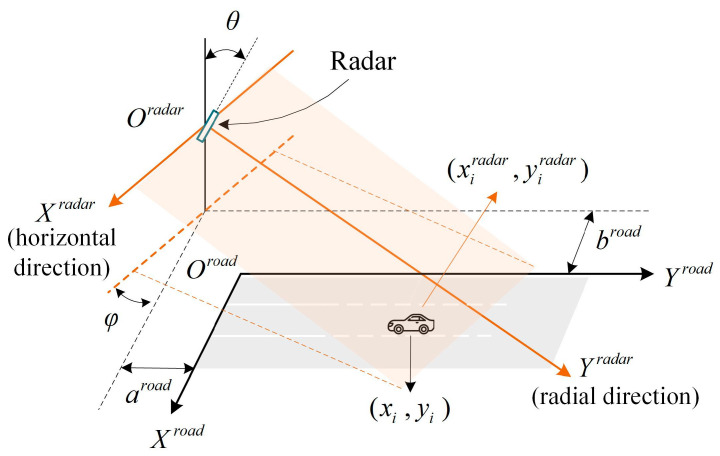
Radar and road coordinate systems.

In this paper, the origin of the radar coordinate system is set as the position where the radar is installed, and the origin of the road coordinate system is set as the joint of the stopping line and the edge of the road. The two coordinate systems are presented in Figure 3.

In Figure 3, θ is the pitch angle between the radar surface and the vertical of the ground. φ is the angle between the *X*-axes or *Y*-axes of the two coordinate systems. $a_{road}$ is the distance between the road coordinate *X*-axis and the stopping line. $b_{road}$ is the distance between the road coordinate *Y*-axis and the edge of the road.

Suppose the position of a vehicle where the radar detects it is $\left(x_{i}^{radar},y_{i}^{radar}\right)$ in $O^{radar}$, the position of the same target in $O^{road}$ can be calculated referring to the geometrical relationship of the two coordinate systems, which is expressed by Equation (1).

(1) $$\left\{ \begin{array}{l} x_{i}^{road}=x_{i}^{radar}\cos\varphi +y_{i}^{radar}\cos\theta \sin\varphi -a_{road}\\ y_{i}^{road}=y_{i}^{radar}\cos\theta \cos\varphi -x_{i}^{radar}\sin\varphi -b_{road} \end{array}\right.$$

where $\left(x_{i}^{road},y_{i}^{road}\right)$ denotes the vehicle position in the road coordinate system.

### 4.2. Trajectory Compensation Methods

#### 4.2.1. Data Missing Identification

When the target is lost and can’t be tracked for a certain period, though actually it is still in the radar’s detection range, the situation is considered as missing data. For the *i*-th vehicle, the data is missed if satisfying the general conditions expressed by Equation (2).

(2) $$y_{i,t_{c}-H\Delta T}^{road}-v_{i,t_{c}-H\Delta T}^{y,road}H\Delta T>0$$

In Equation (2), $t_{c}$ is the current time, ΔT is the radar detection period, and *H* is the threshold of the number of periods. $y_{i,t_{c}-H\Delta T}^{road}$ is the distance of the target at the road coordinate system at time $t_{c}-H\Delta T$ referring to Equation (1) and $v_{i,t_{c}-H\Delta T}^{y,road}$ is the radial speed.

#### 4.2.2. Data Compensation based on Car-Following Behaviors

In this paper, the optimal velocity (OV) model [35], the full velocity difference (FVD) model [36], and the full velocity difference and acceleration (FVDA) model [37] are used to describe the car-following features, and further applied for missing data compensation.

In the OV model, the optimal velocity of the following vehicle *n* is obtained referring to the distance headway, and the motion of the vehicle is described by Equation (3).

(3) $$a_{n}(t)=\alpha \{V[\Delta y_{n}^{road}(t)]-v_{n}^{y,road}(t)\}$$

where α is the sensitivity coefficient of a driver. $\Delta y_{n}^{road}(t)$ is the distance between two vehicles at time *t* and $v_{n}^{road}(t)$ is the speed. V(⋅) is the optimal velocity function calculated by Equation (4).

(4) $$V[\Delta y_{n}^{road}(t)]=\frac{V_{\max}}{2}\{\tanh[\Delta y_{n}^{road}(t)-h_{c}]+\tanh(h_{c})\}$$

In Equation (4), $V_{\max}$ is the maximum speed and $h_{c}$ is the minimum safe distance.

Considering the positive and negative speed differences of the OV model, the FVD model is shown in Equation (5).

(5) $$a_{n}(t)=\alpha \{V[\Delta y_{n}^{road}(t)]-v_{n}^{y,road}(t)\}+\lambda \Delta v_{n}^{y,road}(t)$$

where λ is the sensitivity coefficient of a driver to the velocity difference. $\Delta v_{n}^{y,road}(t)$ is the speed difference between the two vehicles. V(⋅) is the optimal velocity function expressed by Equation (4).

Further, taking the acceleration of the leading car into account, the FVD model is improved, and the FVDA model is presented in Equation (6).

(6) $$a_{n}(t)=\alpha \{V[\Delta y_{n}^{road}(t)]-v_{n}^{y,road}(t)\}+\lambda \Delta v_{n}^{y,road}(t)+\kappa a_{n-1}(t)$$

In Equation (6), κ is the sensitivity coefficient used to describe the driver’s response to acceleration of the leading vehicle acceleration. $a_{n-1}(t)$ is the acceleration value of the leading vehicle. The optimal velocity in the FVDA model is also shown in Equation (4). However, the safe distance in FVDA is a variation parameter directly related to the speed values of the two following vehicles and expressed by Equation (7).

(7) $$h_{c}(t)=\frac{v_{n}^{y,road}{\left(t\right)}^{2}-v_{n-1}^{y,road}{\left(t\right)}^{2}}{2a_{\min}}+\tau v_{n}^{y,road}(t)+l_{n-1}+l_{o}$$

In Equation (7), $a_{\min}$ is the vehicle’s maximum braking deceleration. τ is the reactivity coefficient. $l_{n-1}$ is the length of the vehicle, which is derived from the vehicle type. For FMCW millimeter-wave radar, the vehicle type can be acquired by radar imaging and RCS features. $l_{o}$ is the minimum safe vehicle gap at a stationary state.

Using different car-following models, the acceleration of the vehicle when data missing occurs can be calculated by the leading vehicle, which is directly detected by the radar. Referring to the position and motion status in the former sampling data, the horizontal and radial speed and the horizontal and radial distance of the missing target can be calculated by Equations (8) and (9), respectively.

(8) $$\left\{ \begin{array}{l} v_{n}^{x,road}(t+1)=0\\ v_{n}^{y,road}(t+1)=v_{n}^{y,road}(t)+a_{n}(t)\Delta t \end{array}\right.$$

(9) $$\left\{ \begin{array}{l} x_{n}^{road}(t+1)=x_{n}^{road}(t)\\ y_{n}^{road}(t+1)=y_{n}^{road}(t)+v_{n}^{y,road}(t)\Delta t+\frac{1}{2}a_{n}(t)\Delta t^{2} \end{array}\right.$$

#### 4.2.3. Data Compensation Termination Condition

When the data missing vehicle is re-detected by the radar, the compensation scheme should be terminated to avoid the duplication trajectory of the same vehicle. In this paper, the target similarity index is presented to describe the similarity of the motion features of the compensating trajectory and the actual trajectory, providing a termination scheme for the proposed method.

In the traffic scenario, suppose that vehicles run by lane and the horizontal speed is close to zero with no lane-changing behavior. Hence, the similarity index proposed in this paper mainly considers the vehicle’s radial distance, horizontal distance, and radial speed without the consideration of horizontal speed. Suppose the series of the compensation trajectory acquired by the proposed method in this paper and the actual trajectory obtained by the radar device are expressed by Equations (10) and (11), respectively.

(10) $$Y_{0}=(y_{0}(1),y_{0}(2),\ldots ,y_{0}(i),\ldots ,y_{0}(s))$$

(11) $$Y_{j}=(y_{j}(1),y_{j}(2),\ldots ,y_{j}(i),\ldots ,y_{j}(s)),j=1,2,\ldots ,m$$

In Equations (10) and (11), $y_{j}(i)$ is the vehicle motion vector which consists of horizontal distance, radial distance, and radial speed, as shown in Equation (12).

(12) $$y_{j}(i)=(x^{road},y^{road},v^{y,road})$$

The dimensionless form of Equation (12) is expressed by Equation (13).

(13) $$y_{j}^{}(i)=(\frac{x^{road}}{x_{\max}},\frac{y^{road}}{y_{\max}},\frac{v^{y,road}}{v_{\max}})$$

where $x_{\max}$ and $y_{\max}$ denote the horizontal maximum detection range and radial maximum detection range of the radar. $v_{\max}$ is the maximum road-limited speed.

Then,

(14) $$\Delta y_{{}_{0j}}^{}(i)=\xi \sqrt{{\left[x_{0}^{road}(i)-x_{j}^{road}(i)\right]}^{2}+{\left[y_{0}^{road}(i)-y_{j}^{road}(i)\right]}^{2}}+\eta \left|v_{0}^{y,road}(i)-v_{j}^{y,road}(i)\right|$$

where, $\Delta y_{0j}^{}(i)$ represents the difference between the compensation trajectory and the actual trajectory. ξ and η are coefficients for distance difference and speed difference. Since radar detectability decreases when the vehicle is operating at a certain low-speed, and data is missed at $v_{low}$, ξ and η can be calculated by Equations (15) and (16).

(15) ξ=1−η

(16) $$\eta =\frac{1}{2}\times \frac{\log_{v_{\max}}(v_{j}^{y,road}(i)+v_{low})}{\log_{v_{\max}}(v_{\max}+v_{low})}$$

**Figure 4:**
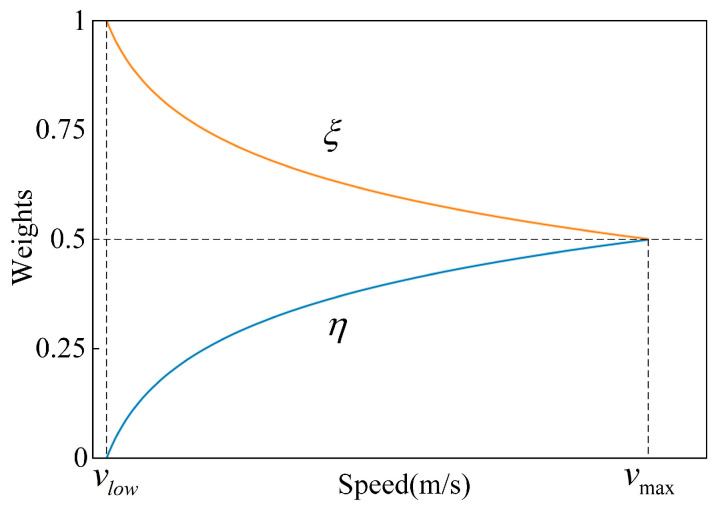
Coefficients of distance difference and speed difference.

The coefficients distributions of distance and speed difference are shown in Figure 4.

The degree of similarity between $Y_{0}^{}$ and $Y_{i}^{}$ is defined as:

(17) $$\gamma (Y_{0}^{},Y_{j}^{})=1-\frac{1}{s}\sum_{i=1}^{s}\Delta y_{{}_{0j}}^{}(i)$$

When the similarity degree is $\gamma (Y_{0}^{},Y_{j}^{})\geq \gamma_{threshold}$, the trajectory data compensation terminal state is reached.

## 5. Experimental Results and Analysis

**Figure 5:**
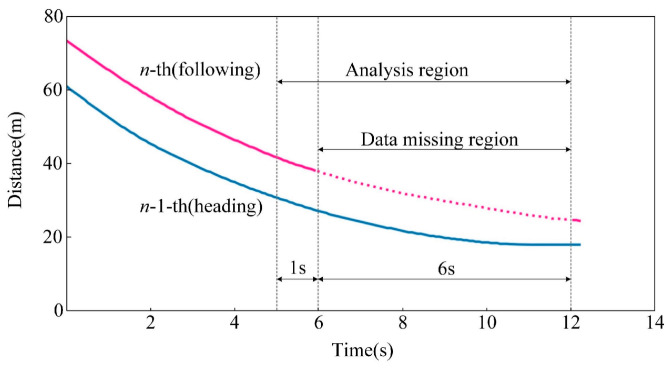
Trajectories of compensation sample.

In this paper, the trajectory data presented in Section 3 is used to verify the performance of the proposed data compensation method. Trajectories of two adjacent vehicles detected by the radar are selected as the sampling data. Meanwhile, part of the trajectory of the following vehicle is rejected artificially to simulate data missing. For the case sample, the last 7s are selected as the analysis region where missing data occurs during the last 6 s. The case trajectories are presented in Figure 5.

The common coefficient values of OV, FVD, and FVDA models are selected in [36,37]. The values of α, λ and κ in them are followed. $V_{\max}$ is the maximum speed limit at the experimental scenario and the rest of the coefficients take commonly used values. The parameters of the OV, FVD, and FVDA models for data compensation in this paper are shown in Table 2.

**Figure 6:**
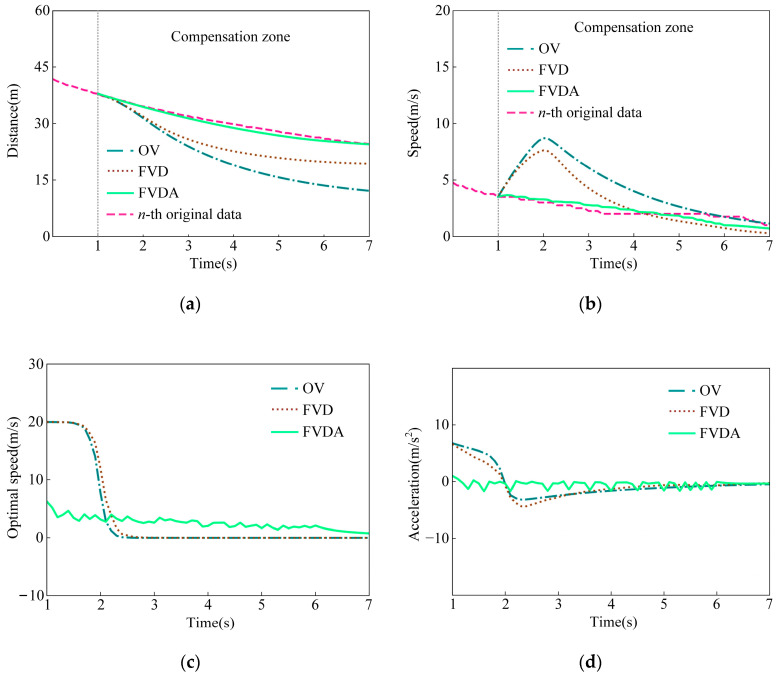
Trajectory compensation results based on different car-following models. (**a**) radial distance compensation results; (**b**) radial speed compensation results; (**c**) radial optimal speed compensation results; (**d**) radial acceleration compensation results.

**Figure 7:**
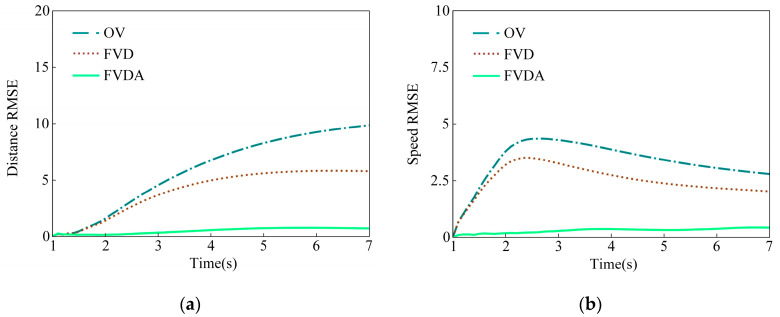
Variations of deviation of different car-following models. (**a**) Radial distance compensation deviations; (**b**) Radial speed compensation deviations.

Using the proposed method based on different car-following models, the trajectory compensation results, including the time-distance curve, time-speed curve, time-optimal speed curve, and time-acceleration curve, are shown in Figure 6. Further, the variations of deviations in distance and speed are shown in Figure 7.

In Figure 6 and Figure 7, the FVDA presents the best performance and the OV model the worst. For the OV model, a sharp acceleration is generated at the beginning of the compensation stage since the speed has not reached the maximum. Simultaneously, the distance between two vehicles exceeds the minimum safety value. Meanwhile, sharp deceleration also occurs when the vehicle’s gap does not satisfy the safe distance, and simultaneously, the speed is greater than the optimized speed during the compensation process. Hence, unreasonable acceleration features are embodied in Figure 6d, inducing that the deviations of the compensation trajectory to the original data are quite high.

In the compensation process using the FVD model, the acceleration behavior is constrained by the speed difference between the two vehicles so that unreasonable acceleration values can be avoided. As shown in Figure 6, the acceleration of the FVD model is smaller than the OV model at the beginning of the compensation process. The reason is that the speed of the leading vehicle is less than the following vehicle, and the speed difference acts as a decelerator. When the vehicle’s gap does not satisfy a safe distance, the following vehicle is more likely to slow down and stop, reducing the probability of collision with the front vehicle. With the FVD model, the compensation trajectory is less than the original trajectory. With the FVD model, the compensation trajectory is less lose to the original trajecetiory.

Referring to Figure 6c, the optimal speed calculated by the FVDA model is less than others because a greater safety distance considering the following vehicle speed is required at the beginning of the compensation stage. Hence, the following vehicle can drive with an appropriate acceleration to avoid emergency braking. During the compensation process, the following vehicle keeps moving at a steady speed. Hence, the trajectory compensation results by the FVDA are more accurate and more reliable than OV and FVD.

To further give a quantitative evaluation of the performance of the proposed method, more samples, including 10 target occlusion missing data trajectories and the 10 low-speed data missing trajectories, are compensated, and the statistical root mean square error (RMSE) is shown in Table 3.

In Table 3, the speed RMSEs of compensation trajectories by the FVDA model and the original speed are less than 1 m/s, and the distance RMSEs are less than 2 m for target occlusion missing data trajectories which present good performance compared with OV and FVD. For low-speed missing data trajectories, the compensation speed and distance RMSEs of the FVDA model are much smaller than the other two models. More statistical results of different missing data trajectories further verify that the FVDA car-following model has marked advantages compared with the OV model and the FVD in compensating speed and distance results.

## 6. Conclusions and Future Work

In this paper, a compensation strategy based on a car-following model is proposed for missing data trajectories collected by millimeter-wave radar. The compensation method provides a systematic solution, including radar coordinate transformation, trajectory compensation, and compensation termination. Experimental results verify the rationality of applying OV, FVD, and FVDA car-following models for the missing data compensation purpose. Besides, the FVDA model achieves the best effect with speed RMSE 0.95 and distance RMSE 1.94 for target occlusion missing data trajectories, and speed RMSE 1.27 and distance RMSE 3.95 for low-speed data missing trajectories.

The future work mainly focuses on two aspects. (1) More car-following methods can be applied to compensate for missing data vehicle trajectories, such as the Gipps, GHR, and IDM models. (2) The proposed compensation method will be verified using different quality data acquired in the complex road environment, for example, trajectory data collected under different weather conditions and other road scenarios.

## Figures and Tables

**Table 1:** Parameters of the installation environment and the road section.

Parameters	Values
Height of the radar	7 m
Pitch angle of the radar surface	12°
Number of lanes	3
Lane width	3.5 m
Distance of the stop line	40 m
Distance of curb line	0.5 m
Frequency range	80–81 GHz
Modulation mode	Frequency-modulated continuous wave
Distance measuring range	0~240 m
Velocity measurement range	−200~250 km/h
Maximum measurement target numbers	128
Detection period	75 ms

**Table 2:** Parameters of the car-following models.

Parameters	Values
α	0.41
$V_{\max}$ (m/s)	20
$h_{c}$ (m)	7.5
λ	0.5
κ	0.5
$a_{\min}$ (m/s^2^)	5
τ	1
$l_{n-1}$ (m) of light vehicle	5
$l_{n-1}$ (m) of heavy vehicle	7.5
$l_{o}$ (m)	2.5

**Table 3:** Statistical deviations of different data missing trajectories based on different car-following models.

Error Type	Time(s)	Target Occlusion Data Missing Trajectory			Low-Speed Data Missing Trajectory		
		OV	FVD	FVDA	OV	FVD	FVDA
Speed RMSE	1	1.69	1.29	0.78	3.12	2.34	0.98
	2	2.72	1.86	0.79	5.04	3.27	1.25
	3	3.10	1.98	0.83	5.80	3.39	1.41
	4	3.21	2.06	0.87	6.10	3.28	1.42
	5	3.12	2.09	0.92	5.97	3.18	1.35
	6	3.04	2.04	0.95	5.60	3.00	1.27
Distance RMSE	1	0.97	0.82	0.45	1.33	1.04	0.52
	2	2.70	1.97	0.69	4.49	3.12	1.24
	3	4.76	3.21	0.97	8.30	5.26	2.10
	4	6.63	4.24	1.28	12.14	7.02	2.94
	5	7.89	5.03	1.63	15.67	8.43	3.58
	6	8.54	5.56	1.94	18.57	9.54	3.95

## Data Availability

Not applicable.
